# The Full Spectrum of Clinical Ethical Issues in Kidney Failure. Findings of a Systematic Qualitative Review

**DOI:** 10.1371/journal.pone.0149357

**Published:** 2016-03-03

**Authors:** Hannes Kahrass, Daniel Strech, Marcel Mertz

**Affiliations:** 1 Institute for History, Ethics and Philosophy in Medicine, Hannover Medical School, Hannover, Germany; 2 Center for Ethics, University Hospital Cologne, Cologne, Germany; Sao Paulo State University, BRAZIL

## Abstract

**Background:**

When treating patients with kidney failure, unavoidable ethical issues often arise. Current clinical practice guidelines some of them, but lack comprehensive information about the full range of relevant ethical issues in kidney failure. A systematic literature review of such ethical issues supports medical professionalism in nephrology, and offers a solid evidential base for efforts that aim to improve ethical conduct in health care.

**Aim:**

To identify the full spectrum of clinical ethical issues that can arise for patients with kidney failure in a systematic and transparent manner.

**Method:**

A systematic review in Medline (publications in English or German between 2000 and 2014) and Google Books (with no restrictions) was conducted. Ethical issues were identified by qualitative text analysis and normative analysis.

**Results:**

The literature review retrieved 106 references that together mentioned 27 ethical issues in clinical care of kidney failure. This set of ethical issues was structured into a matrix consisting of seven major categories and further first and second-order categories.

**Conclusions:**

The systematically-derived matrix helps raise awareness and understanding of the complexity of ethical issues in kidney failure. It can be used to identify ethical issues that should be addressed in specific training programs for clinicians, clinical practice guidelines, or other types of policies dealing with kidney failure.

## Introduction

Chronic kidney failure is the gradual loss of kidney function. It can progress to end-stage kidney failure, which is fatal without artificial filtering (dialysis) or a kidney transplant. The care of patients with kidney failure involves diverse ethical challenges. For example, many patients with chronic kidney failure need to decide whether to begin or stop dialysis therapy. Dialysis may offer considerable benefits (e.g., be life sustaining), but also entails major burdens (e.g., is very restrictive and time consuming). Balancing these benefits and side-effects for different stages of the disease involve complex value judgments. Further ethical issues might come with heterogeneities of referral criteria for transplantation or financial conflicts of interest that could intervene in a sound inter-professional management of patients with chronic kidney failure.

Several papers and books explicitly address ethical issues in kidney failure that include but also go beyond issues in dialysis, as well as case books covering a wide spectrum of ethical issues in kidney patients (e.g., “Ethics and the kidney” [1] or “Legal and Ethical Concerns in Treating Kidney Failure” [2]).

Despite the range of ethical issues in health care for patients with chronic kidney failure, major professional organizations and their clinical practice guidelines (CPGs) mostly focus on the ethical issues of withdrawing dialysis. For example, the U.S. Renal Physicians Association (RPA) and the American Society of Nephrology (ASN) issued a CPG called “Shared Decision Making in the Appropriate Initiation of and Withdrawal from Dialysis” [3], while the Renal Association in the United Kingdom issued the guideline “Planning, Initiating and Withdrawal of Renal Replacement Therapy” [4].

These guidelines are meant to improve standards of clinical competence and professionalism by referring explicitly to evidence-based information on benefits and harms [5]. Jean Holley et al. demonstrated their effects on nephrologists’ end-of-life decision-making [6]. It seems obvious that withdrawing dialysis is a recurrent question in nephrology, and so it seems advisable to address this ethical issue in CPGs. However, even if there are rationales for a particular selection of ethical issues or for focusing on specific ethical issues in a CPG, this can lead to bias or at least incomplete support for medical professionalism, because health care professionals lack awareness of other ethical issues in the same clinical care domain. A meaningful countermeasure here is to make the full spectrum of relevant ethical issues transparent and to be explicit about the rationale for focusing on specific ethical issues. A systematic review of relevant literature can derive such a full spectrum of ethical issues in the care of patients with kidney failure. Such a full spectrum of disease-specific ethical issues (DSEIs) was published recently in the context of dementia care [7]. Another study demonstrated that national CPGs for dementia care vary substantially as to which ethical issues they address [8].

In our review, we aimed to identify the full spectrum of ethical issues that can arise for patients with kidney failure. For this, we reviewed literature (including journal articles, reports and books) published between 2000 and 2014.

Our assessment is purely descriptive. The identified ethical issues are not valued or ranked. This means that our review does not make any normative recommendations, i.e. how one should deal with the ethical issues. However, the full spectrum of DSEIs in kidney failure could underpin a more systematic and transparent development of CPGs, national and regional care plans, continuing medical education (CME) programs and capacity-building activities in this regard.

## Method

### Literature search

The literature search methods are closely based on those of Strech et al. [7], which describe the methodological approach in detail. We searched Medline using Boolean operators (AND and OR) with variations of the search terms ‘ethics’ and ‘kidney disease’ (for more details on the search strategy see S1 Table). Language restrictions for the search were English and German. The search was done in January 2015 and covered the last 15 years (1st January 2000 to 31st December 2014). We examined the full text if a publication was considered relevant on the basis of its title and abstract. The inclusion was solely based on the pre-established criteria (see below). In accordance with our claim of a descriptive review and the general problem of a standardized quality appraisal of normative–ethical (e.g. philosophical) literature, the methodological quality of the literature was not assessed.

We also searched in Google Books with the search string “Kidney failure AND ethics”. We accessed Google Books and used the default settings (sort by relevance). We applied the same eligibility criteria and procedures for the journal articles, book chapters and the monographs. Because there is no function for language restriction in Google Books, we manually filtered by language. Because of the vast number of hits (14,600), and because Google Books lists results in order of diminishing relevance, we focused on the first 50. We regard the ordering by relevance to have had face validity, because we found among these first 50 hits many textbooks and monographs that dealt with kidney failure and ethics.

Because of a lack of standardization of key words for bioethics in databases and varying definitions of ethics in the literature, we applied additional search techniques to ensure that we covered all relevant literature. We therefore carefully screened all the references of already- included literature for additional relevant publications.

### Definition of “disease-specific ethical issue” (DSEI)

We base our definition of “disease-specific ethical issue” (DSEI) on an ethical approach called principlism [9]. Principlism, which is used as a basis by many ethical and medical professionalism frameworks [10], is based on four “mid-range” principles: beneficence, non-maleficence, respect for autonomy, and justice. Each principle formulates obligations that are valid prima facie. This means that they must be followed in a particular case unless there is a conflict with another obligation that is of equal or greater weight. The principles themselves, though, are only general orientations. In a specific case, they require further content, have to be put in context and have to be balanced against one another if they conflict.

A DSEI, therefore, can arise when one or more (contextualized) principles have been inadequately considered. For example, most text passages that were subsumed under our DSEI 2 *Dialysis* dealt with avoidable issues of neglecting the burdens that result from dialysis, which would go against the principle of non-maleficence (see DSEI 2 in Table 1). On the other hand, a DSEI might arise because of conflicts between two or more (contextualized) principles. This is for example the case when secondary interests (financial or non-financial) conflict with the primary interest of balanced provision of information and therapeutic options (see DSEI 3.1a–c *Conflict of Interest* in Table 1).

**Table 1:** The spectrum of disease-specific ethical issues (DSEIs) issues in kidney failure.

**1 Diagnosis, prognosis and medical indication**			
First and second-order issues	Original wording (examples)	References	Principle
1.1 Diagnosis			
1.1.a Heterogeneous criteria to withhold (not to start) dialysis	Nephrologists withheld dialysis from 25 of 357 (7%) ESRD patients compared with 42 of 193 (22%) withheld by primary care physicians (P F 0.001). [13]	[1, 6, 11, 13, 14, 17, 20–39]	Justice
1.1.b Heterogeneous criteria to withdraw dialysis	Academic nephrologists who had received education in the ethics and law of stopping dialysis withdrew it from a greater percentage of patients than those in private practice (12% v 6%; P 5 0.009). [13]	[1, 6, 13, 17, 26–28, 30–33, 35, 37–50]	Justice
1.1.c Heterogeneous referral criteria for transplantation	There are emerging data that referrals for renal transplantation, the treatment of choice for ESRD, are made less often from for-profit than from not-for-profit dialysis providers. [12]	[12, 14, 23, 24, 27, 38, 51, 52]	Justice
1.1.d Heterogeneous referral to renal specialist	Additionally, there is substantial evidence that patients needing dialysis are being referred to nephrologists too late and that access to renal replacement therapy is related to the patient’s sociodemographic characteristics. [24]	[24, 33, 53]	Justice
1.2 Prognosis			
1.2.a. Heterogeneity in the medical assessment (prognosis)	Table 1 shows how the time to referral to a nephrologist varied. Among the most notable findings are the higher hazard ratios (indicating a tendency to earlier referral) for diabetic patients, for women, for younger patients and for patients from more deprived areas, after adjusting for the other relevant variables in the Cox analysis. [24]	[1, 20, 21, 29, 34, 38, 40, 54–56]	Justice
1.2.b Various concepts of futility	Consideration of futility during EOL did not receive adequate attention in this unit, which incurred an additional human and material burden. [40]	[1, 14, 38, 40, 57–60]	Non-maleficence
**2 Dialysis**			
2.1 Adequate consideration of physiological (“somatic”) side-effects and treatment burden (e.g., pain)	There are increasingly more situations in which we may doubt its salutary effects and conclude that it is not always adequate to fulfill the real objective of medicine: providing care, without necessarily curing. [25]	[1, 25, 31, 37, 38, 52, 61–63]	Non-maleficence, beneficence
2.2 Adequate consideration of psychological side-effects and treatment burden (quality of life)	Significant differences were found between nurses’ and patients’ ratings of QoL, health status, functional status, outlook, and support.[31]	[1, 14, 20, 23, 31, 37–39, 42, 52, 58, 62–69]	Non-maleficence, beneficence
2.3 Adequate consideration of (inter-) social side-effects and treatment burden (quality of life)	Access to work-friendly treatments with less rigid schedules, such as transplant, PD, or some form of HHD, may help the 50% of incident patients each year who are of working age keep their jobs. [23]	[23, 31, 37, 61, 62]	Beneficence, non-maleficence
**3 Information and disclosure**			
3.1 Adequately managing conflicts of interests			
3.1a financial COI of the provider (institution)	With the change in ownership of dialysis facilities comes the obvious corollary that the major responsibility of the new corporate owners is to shareholders, not to patients or their physicians. [12]	[1, 11, 12, 37, 61, 70–73]	Respect of autonomy, justice, non-maleficence, beneficence
3.1b financial COI of the physician	Physicians who are more knowledgeable about medical law are more likely to withdraw patients from life-sustaining treatments [. . .] In addition, while it may not be decisive, there is a possible third reason. The compensation of academic and community nephrologists in the United States differs; community nephrologists are more directly impacted financially by decisions to stop dialysis. [13]	[1, 12, 13, 37, 61, 72]	Respect of autonomy, justice, non-maleficence, beneficence
3.1c other (non-financial) COI	A 58 year old physician, one of the pioneers in introducing peritoneal dialysis for chronic renal failure, consistently coerces his patients into maintenance peritoneal dialysis rather that maintenance hemodialysis. By contrast, another nephrologist at the same facility, who is expert in hemodialysis, does not even consider peritoneal dialysis in the choices presented to newly uremic patients. At some facilities, home hemodialysis is not mentioned, while at a few others, home hemodialysis is promoted as the therapy most likely to permit long survival during maximized rehabilitation.[14]	[14]	Respect of autonomy, justice, non-maleficence, beneficence
3.2 Adequate patient information (amount of information, setting)	The low PD utilization rate in most countries indicates that many patients were either not given true free choice for PD or they were not given unbiased information and education before making a choice. [11]	[1, 11, 14, 23, 58, 61, 67, 74]	Respect of autonomy, non-maleficence
3.3 Adequate support of positive (realistic) beliefs	The patient’s health care and QoL goals should be the main focus when considering whether or not to start HD in the frail elderly patient. Physicians must be careful not to encourage unrealistic expectations of the benefits of dialysis and be frank about the associated risks and modest impact on survival. Patients with ESRD also need to have a realistic expectation of how renal replacement therapy will impact their daily life. [75]	[11, 20, 23, 30, 38, 58, 66, 67, 69, 75–78]	Respect of autonomy, non-maleficence, beneficence
**4 Decision-making & consent**			
4.1 Adequate consideration of patients preferences	two-thirds of patients with chronic kidney disease (CKD) indicated that they chose HD over supportive care because it was their physician’s (52%) or family’s (14%) wish, and 61% of these dialysis patients regretted having started HD. [75]	[11, 20, 30, 35, 38, 42, 46, 47, 58, 61, 63, 64, 75, 79–81]	Respect of autonomy, beneficence
4.2 Adequate assessment of the cognitive abilities	Surveys and responses to hypothetical scenarios have repeatedly shown that a patient’s ability to relate and respond to the world is the most important factor in decisions to initiate and withdraw dialysis. [53]	[53, 58, 82]	Respect of autonomy, non-maleficence
4.3 Professional distress over patient’s decision to discontinue dialysis	Several studies show that physicians experience ethical dilemmas concerning the withholding or withdrawing of life-sustaining treatments […]. Withdrawal of treatment may be experienced as unethical as physicians have a responsibility and a duty to save life. [83]	[47, 58, 62, 83, 84]	Respect of autonomy
**5 Social and context-dependent aspects**			
5.1 Adequately dealing with relatives	A review of current literature was undertaken and revealed a paucity of information in regard to palliation in those with end stage renal disease who had discontinued dialysis. The fear of dying, pain, suffering, and abandonment that a patient and/or their family may perceive as being associated with death may create barriers to decisions to discontinue with dialysis treatments. Therefore health care personnel should provide information with honesty to allow patients to predict their quality of life and death. [42]	[14, 31, 38, 42, 83, 85–88]	Beneficence
5.2 Disregarding the interest or needs of particular groups (e.g. gender or race) (discrimination)	Statistically significant differences were found with respect to the length of stay for discharge status and gender; and with respect to costs for surgery versus no surgery and gender. Significant differences were also found between discharge status and gender, age, and cardiovascular surgery. [40]	[37, 40, 51, 87, 89–91]	Justice
5.3 Fair rationing	Decisions regarding the allocation of limited medical resources such as the Medicare budget should consider ethically appropriate criteria including likelihood of benefit, urgency of need, change in quality of life, duration of benefit, patient selection, equitable distribution, and the amount of resources required. In examining the evidence base on daily dialysis according to these ethical criteria, we find that there are not yet sufficient grounds to recommend funding of daily dialysis by the Medicare ESRD Program. [92]	[11, 37, 58, 64, 89, 92–96]	Justice
**6 Care process & process evaluation**			
6.1 Adequate advance care planning (advance directives)	Since there is a presumption in favor of continued life-sustaining treatment for patients who cannot and have not expressed their wishes, the patient’s right to stop dialysis in certain situations is usually difficult to achieve unless patients have explicitly stated their preferences in advance in an oral or written advance directive or have named a proxy to speak for them. [13]	[13, 47, 49, 61, 83, 89]	Respect of autonomy, beneficence, non-maleficence
6.2 Dealing with lack of evidence (e.g. on patients’ preferences and attitudes)	Hemodialysis is associated with a high rate of complications and has not been shown to prolong life in cirrhotic patients with acute renal failure (ARF), but has not been carefully examined in those with CKD. [65]	[20, 53, 64, 65, 77, 92, 97]	Beneficence, non-maleficence
6.3 Insufficient advanced training and CME (e.g. to promote DMC and self-reflection)	Expanding the training of our nephrologists and the ESRD/ nephrology multidisciplinary care team to include communication, prognostication, and end-of-life care may help bridge that gap. [75]	[13, 25, 28, 58, 75, 98–101]	Beneficence, non-maleficence
**7 Subgroups with special concerns**			
7.1 Patients with insufficient decision-making capacity (e.g. mentally ill patient)	Medical co-morbidities (ESRD) are very common in patients with psychiatric conditions. Although respecting one’s autonomy to make treatment decisions is the ethical default position, the capacity to make such decisions may need to be assessed, especially when patients are in relapse of their psychiatric condition, and/or when the decisions made are high-risk and possibly fatal. [102]	[14, 28, 29, 38, 41, 47, 89, 102]	Respect of autonomy, beneficence
7.2 Considering poor prognosis and severe co-morbidities in treatment decisions	Interestingly, the burden of comorbid conditions was comparable in the dialyzed and conservatively treated groups, suggesting that comorbidity per se was either not primary in decision making or considered ‘‘pejoratively in the context of late referral, poor functional status, or social isolation.’ [53]	[14, 20, 38, 53, 63, 67, 86, 89, 93, 103–105]	Beneficence, non-maleficence
7.3 Dealing with non-compliant patients	Is there a limit to the number of times a noncompliant but competent patient is entitled to emergent dialysis to treat complications resulting directly from his own irresponsible and inconsiderate behavior? [97]	[14, 30, 38, 58, 63, 66, 89, 97, 106–108]	Justice, beneficence, non-maleficence
7.4 Balancing risks and benefits for vulnerable groups (e.g. pregnant woman, neonates and elderly)	From the pediatric nephrologist's perspective, renal transplantation is almost always presented as the optimal long-term solution […]. Children who received a kidney transplant before 18 years of age and who maintained graft functioning for at least 10 years had a favorable social and professional outcome (Morel et al. 1991). This expert bias generates pressure on apprehensive parents in favor of a decision that may overlook the child and family’s readiness for the procedure. [89]	[1, 6, 22, 31, 34, 41, 53, 61, 79, 89, 103, 109–117]	Justice, beneficence, non-maleficence

### Eligibility criteria

For our purpose of a spectrum of clinical ethical issues, we used the following criteria for inclusion of a publication in our review: (I) it had to describe a DSEI related to clinical care of kidney failure patients; (II) it is possible for individual care-givers or care institutions to deal with the particular DSEI, i.e. dealing with the DSEI is not dependent on health policy or political decision-making (therefore we excluded, for example, transplantation issues such as those related to living donors; the only transplantation issue we included was the decision to put a particular patient on the transplantation list); (III) the publication was not only focused on ethical issues when researching kidney failure (issues of research ethics); and (IV) the publication had to be a peer-reviewed journal article, a scientific book (monograph, textbook, edited volume…) or a national-level report. The methodological quality, beyond the fact that the paper was identified in scientific databases and published in peer-reviewed journals, did not serve as a criterion of eligibility criteria, as the quality of a paper was irrelevant to the purpose of the review: to identify the spectrum of clinical ethical issues.

### Extraction, analysis and synthesis of disease-specific ethical issues

Our aim was to develop a qualitative framework of DSEIs (that is, a full spectrum of DSEIs) that optimally accommodated the ethical issues mentioned in the publications analyzed. Therefore, the 95 articles included were grouped into three clusters of 45, 30 and 20 publications. All publications that at initial inspection appeared to be more detailed and comprehensive were purposively put in the first cluster. One author (HK) identified, extracted and compared paragraphs which mentioned ethical issues from the 45 articles. A second author (MM) independently checked the identification and the matching of the text passages that addressed DSEIs. Both authors were trained in qualitative text analysis and normative methods and had already applied these methods in reviewing DSEIs [7]. The result was a first version of the DSEI spectrum. The second (n = 30) and third (n = 20) clusters were then used to check theoretical saturation of the DSEI spectrum. Theoretical saturation implies that no new categories will be generated. The books were grouped and analyzed in two clusters (n = 5 and n = 6). Then, we categorized the issues in consensus discussions with the third author (DS). We built first-order (broad) and second-order (narrow) categories for DSEIs which captured similar ethical issues mentioned in different papers.

Further, we noted against each second-order DSEI the underlying general ethical principle [9]. One author (HK) proposed such a corresponding principle, and the other authors (MM/DS) checked for consistency. The researchers were able to overcome all initial ambiguities and differences in the analysis and synthesis of DSEIs in open discussion.

## Results

### References and Journals

From our Medline search, 84 references out of 233 were finally included. Additionally, we identified 11 relevant references by reference tracking (published between 1992 and 2014). The original documents in the final sample were published in 54 different journals with the most frequently represented being *Seminars in Dialysis* (n = 7, 7%), *Nephrology News & Issues* (n = 7, 7%), *Advances in Chronic Kidney Disease* (n = 5, 5%), *Clinical Journal of the American Society of Nephrology* (n = 5, 5%), and *American Journal of Kidney Diseases* (n = 5, 5%).

The final sample originated from nine different medical fields, dominated by Nephrology (n = 62, 65%) and followed by General Medicine (n = 12, 13%) and Ethics (n = 8, 8%). Others were published in journals for Health Care Policy, Medical Science, Nursing, Palliative Care, Pediatrics, Psychology, and Social Work. The majority were published in English (96%).

**Fig 1:**
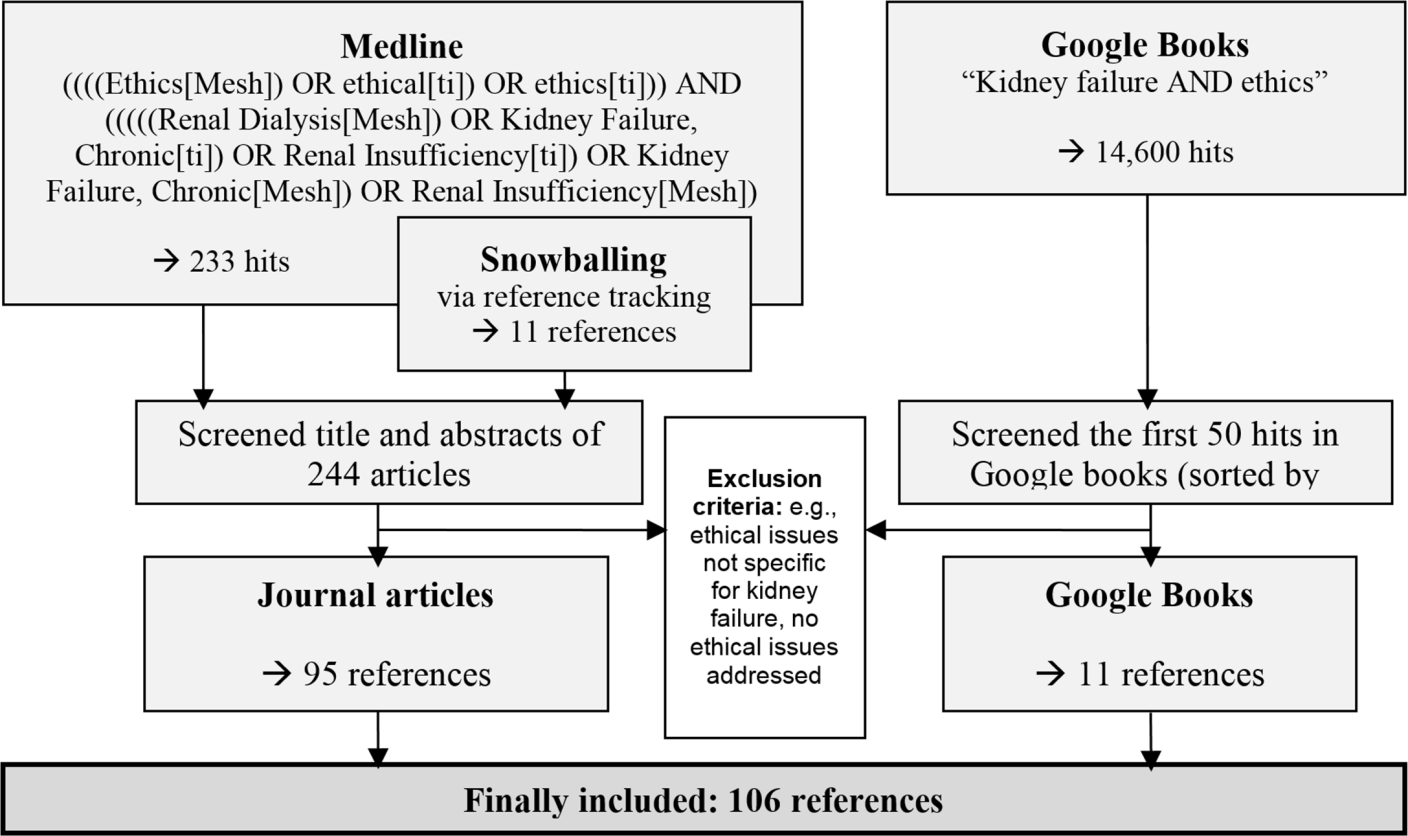
Flow diagram for inclusion/exclusion of references.

Via Google Books we retrieved 15 relevant books, of which 11 were included in our analysis. The books addressed normative issues in kidney failure (n = 3), normative issues in medical care–including a chapter on kidney failure (n = 3), health care in kidney failure–including a chapter on ethical issues (n = 5), and health care in chronic disease–including ethical issues in chronic kidney failure (n = 1) (Fig 1).

### Disease-specific ethical issues (DSEIs)

We were able to identify 27 DSEIs for kidney failure, grouped into seven major thematic categories: (I) Diagnosis, prognosis and medical indication; (II) Dialysis; (III) Information and disclosure; (IV) Decision-making and consent; (V) Social and context-dependent aspects; (VI) Care process and process evaluation; and (VII) Subgroups with special concerns. Most of these major categories were further differentiated into first and second-order subcategories. For example, we subdivided the fourth category “Decision-making and consent” into “4.1. patients preferences”, “4.2. assessing cognitive abilities” and “4.3. professional distress over patient’s decision to discontinue dialysis” (Table 1).

This set of 27 DSEIs in kidney failure is based upon 286 text passages identified by our systematic review. The number supporting each individual issue varies from one to 26 references (the corresponding numbers of sources for each DSEI are stated in Table 1).

For a text passage to be included, it was not necessary that it explicitly address the ethical issue. It was sufficient that it could be interpreted as implicitly addressing the ethical issue, e.g., **“**The low PD utilization rate in most countries indicates that many patients were either not given true free choice for PD or they were not given *unbiased information* and education before making a choice” [11] (text example to substantiate DSEI 3.1 “Adequate patient information”) and “There are emerging data that referrals for renal transplantation, the treatment of choice for ESRD, are made less often from for-profit than from not-for-profit dialysis providers” [12] or “Nephrologists withheld dialysis from 25 of 357 (7%) ESRD patients compared with 42 of 193 (22%) withheld by primary care physicians” [13]. Here, the first example implicitly refers to a conflict with respect for patient autonomy, and the second and third to a conflict with the principle of justice (Table 1).

## Discussion

This systematic literature review identified and synthesized the full spectrum of 27 DSEIs in kidney failure based on 106 articles published between 1992 and 2014 in 54 different journals and 11 books. Many identified DSEIs cover ethical issues beyond the prominent question of withdrawing dialysis (Table 1). While this paper presents the full spectrum of 27 DSEI in a descriptive way, an in-depth analysis or normative evaluation of the identified DSEIs is beyond the scope of this paper. The following discussion, therefore, focuses on the validity and potential uses of the full spectrum of DSEIs presented here.

To increase the utility and applicability of the DSEI spectrum in clinical care, patient information, clinical practice guidelines, and policies, the first six main categories correspond to common elements of clinical practice: (I) Diagnosis, prognosis and indication, (II) Therapy–here Dialysis, (III) Information and disclosure, (IV) Decision-making process and consent, (V) Social and context-dependent aspects and (VI) Care process, its evaluation and consideration by health professionals. This is complemented by a seventh main category for issues that could not be assigned to above-mentioned categories: (VII) Patient subgroups with special concerns. This overarching categorization is based on previous work by the same research group [7].

### Interpretation of the validity and relevance of DSEIs

Two DSEIs were mentioned in many different papers: “heterogeneous criteria to withhold (not to start it) OR withdraw dialysis” (mentioned in 26 papers). Others were mentioned by few sources: “non-financial conflicts of interest” (n = 1), “heterogeneous referral criteria for transplantation” (n = 3), and “adequate assessment of the cognitive abilities” (n = 3). This raises the question of what conclusions may be drawn from this information. Numerous references for one DSEI could suggest that this DSEI is of high importance. Conversely, little attention paid in the literature to an issue could indicate its secondary importance. However, the fact that there are few references for a DSEI could also be ascribed to its complexity or controversial nature. Also, recent issues with high relevance for current practice might be addressed less often in the literature. A DSEI having many references may just mirror the fact that it is a standard topic in (current) academic discourse (and, possibly, with funding agencies), which may not necessarily correspond to the issue’s clinical ethical importance.

Therefore, normative conclusions should not be lightly drawn from the number of references to a DSEI. It is fair to say, then, that one advantage of trying to establish a full spectrum of DSEIs for kidney failure is the inherent methodological tendency to avoid either excluding a DSEI because it is rarely addressed in the literature, or regarding a DSEI as important because it is covered extensively.

Relevance assessments could also be misled by the variety of text passages that address DSEIs. An ethical issue (e.g., neglecting the principle of justice) can be addressed by referring to statistical data, for example: “Nephrologists withheld dialysis from 25 of 357 (7%) ESRD patients compared with 42 of 193 (22%) withheld by primary care physicians (P F 0.001)” [13] and: “There are emerging data that referrals for renal transplantation, the treatment of choice for ESRD, are made less often from for-profit than from not-for-profit dialysis providers” [12]. However, it can also be addressed in a narrative way, for example: “A 58 year old physician, one of the pioneers in introducing peritoneal dialysis for chronic renal failure, consistently coerces his patients into maintenance peritoneal dialysis rather than maintenance hemodialysis. By contrast, another nephrologist at the same facility, who is expert in hemodialysis, does not even consider peritoneal dialysis in the choices presented to newly uremic patients.” [14]

While narrative description of DSEIs is associated with qualitative research, case studies, or even anecdotal evidence based on the author’s own observation, statistical description of DSEIs is clearly associated with quantitative research. As with the prevalence of references to a DSEI, one should be careful when ascribing relevance or precedence to a DSEI solely based on the kinds of description found in the scientific or scholarly literature. This is especially important against the backdrop of evidence-based approaches. These may devaluate narrative descriptions because of established “levels of evidence” [15], where evidence from, e.g., case studies, are ranked as very weak evidence. In ethics, however, even a case study can show what ethical challenges or conflicts of ethical principles can occur in a specific situation. Nonetheless, the distinction between narrative and statistical description may trigger a discussion about the methodological quality of DSEI descriptions, as well as about what level of confidence we should have in the literature from which we obtain our DSEIs.

### Uses of the DSEI spectrum

Such a systematic review of ethical issues in kidney failure can serve several purposes. First, it can raise awareness about the variety of ethical issues and the complexity of ethical conduct in the care of patients with kidney failure, provided that the spectrum is well presented (see above).

Second, it can form a basis for the systematic development of informational and training materials for health professionals, relatives, patients, or the wider public. It is hardly possible (and probably not necessary) to address all issues exhaustively. Thus, prioritization procedures with (clinical/practical/political) relevance as core criteria are important. The spectrum could be an objective ground for discussions of the relevance (e.g., from a clinical or policy perspective) of certain ethical issues. As mentioned earlier, our spectrum does not allow conclusions to be drawn directly about the relevance of a particular DSEI. Nevertheless, as the relevance of a certain issue strongly depends on the context, the spectrum could be a valuable starting point for researching that context, and thus, the relevance of specific issues. For clinical purposes, the views of clinicians and/or patients are crucial (for example, Grönlund et al. performed narrative interviews with nurses and physicians which allowed them to identify relevant issues in this context [16, 17]). Thus, our findings could make the process of identifying and agreeing on key DSEIs systematic and transparent.

Other possible fields of application include guideline (CPG) development, and other local and national polices for clinical care and continuing medical education (CMA) programs. We would like to emphasize that current CPGs are developed by and with clinical and other experts and hence will include a relevant sample of ethical issues. The achievements of CPGs are laudable from an ethical perspective. Here, we propose the application of the presented full spectrum of DSEIs to future development or revision of CPGs addressing kidney failure.

The spectrum includes, in addition, the original documents from which the DSEIs emerge, as well as the relevant underlying basic ethical principles (Table 1). This information is useful in the process of drafting recommendations or developing strategies to deal with the ethical issues presented, e.g., by helping to raise awareness of the respective function of (I) the general ethical principles (e.g., “respect the autonomy of the patient”), and (II) disease and care-setting–specific adaptations (e.g., “Since there is a presumption in favor of continued life-sustaining treatment for patients who cannot and have not expressed their wishes, the patient’s right to stop dialysis in certain situations is usually difficult to achieve unless patients have explicitly stated their preferences in advance in an oral or written advance directive or have named a proxy to speak for them.”) [13], see DSEI 6.1 in Table 1). However, one should bear in mind that the quality and relevance of particular issues have not been determined. Thus, the spectrum displayed in Table 1 is of a purely descriptive nature.

Finally, one could use the comprehensive spectrum to assess the coverage of relevant clinical ethical issues in existing practice guidelines or CMA programs. We have demonstrated such an assessment in the case of dementia guidelines [8].

### Limitations, open questions and need for further research

One limitation of this systematic review of DSEIs might be seen in the fact that we restricted our search to Medline and Google Books. We restricted our search in this way for two main reasons: first, and most important, we reached theoretical saturation for the first and second-order categories of DSEIs after assessing the 106 references retrieved for Medline, Google Books, and from reference checks. Second, the characteristics of publications included in this systematic review (Table 1) demonstrate that the 106 references covered journals and books from all relevant fields.

Another limitation is that the literature search in databases was restricted to the last 15 years. The period was chosen for pragmatic reasons. However, we retrieved further papers up to the year 1992 by additional reference checks. We further validated the results by checking for theoretical saturation.

A useful complement to the systematic review would be to interview experts such as bioethicists, patients and medical staff from a dialysis unit. Individual stakeholder views have been included in our review based on the nurses’ and physicians’ narratives from Catarina Grönlund et al. [16, 17]. These approaches are especially suited to obtain narrative descriptions of DSEIs, and to avoid the problem that the voices of patients and other stakeholders can remain unheard when it comes to the identification, definition and selection of relevant ethical issues [18, 19].

## Conclusion

The care of people with kidney failure unavoidably involves ethical issues. Dealing adequately with these issues is a significant element of medical professionalism. A major prerequisite for doing so is an unbiased awareness of the scope, diversity and complexity of DSEIs in kidney failure. While much has been written on ethical issues in kidney failure, the DSEI spectrum presented in this paper gives a more comprehensive and transparent account of all relevant ethical issues. Further, the supplementary information (ethical principles and references) could be a good starting point for shaping educational material or developing practice guidelines on kidney failure that include explicit information on relevant clinical ethical issues. The authors received no additional funding for conducting this review, and have no relations to private companies. There are no conflicts of interest to declare.

## Supporting Information

S1 Table Details on the search strategy. (DOCX)

## References

[1] Levinsky NG. Ethics and the kidney Oxford; New York: Oxford University Press; 2001.

[2] Friedman EA. Legal and ethical concerns in treating kidney failure case study workbook. 2000.

[3] RPA. Shared decision-making in the appropriate initiation of withdrawal from dialysis Rockville (MD): Renal Physicians Association (RPA); 2010.

[4] Warwick G, Mooney A, Russon L, Hardy R. Planning, Initiating and Withdrawal of Renal Replacement Therapy UK Renal Association 2014. 10.1159/000328069 21555896

[5] IOM. Clinical practice guidelines we can trust (IOM) IoM, editor. Washington (D.C.): National Academies Press; 2011.

[6] Holley JL, Davison SN, Moss AH. Nephrologists’ Changing Practices in Reported End-of-Life Decision-Making. Clinical Journal of the American Society of Nephrology. 2007;2(1):107–11. 10.2215/cjn.03080906 17699394

[7] Strech D, Mertz M, Knuppel H, Neitzke G, Schmidhuber M. The full spectrum of ethical issues in dementia care: systematic qualitative review. Br J Psychiatry. 2013;202:400–6. Epub 2013/06/05. 10.1192/bjp.bp.112.116335 . 23732935

[8] Knüppel H, Mertz M, Schmidhuber M, Neitzke G, Strech D. Inclusion of ethical issues in dementia guidelines: a thematic text analysis. PLoS Med. 2013;10(8):e1001498 Epub 2013/08/24. 10.1371/journal.pmed.1001498 23966839 PMC3742442

[9] Beauchamp TL, Childress JF. Principles of biomedical ethics New York: Oxford University Press; 2009.

[10] Foundation A. Medical professionalism in the new millennium: a physicians' charter. The Lancet. 2002;359(9305):520–2. doi: 10.1016/S0140-6736(02)07684-5. 11853819

[11] Lo WK. Absolute free choice for dialysis modality selection—is it possible? Perit Dial Int. 2009;29(2):142–3. 19293348

[12] Bennett WM. Ethical conflicts for physicians treating ESRD patients. Seminars in dialysis. 2004;17(1):1–3. Epub 2004/01/14. . 14717800 10.1111/j.1525-139x.2004.17102.x

[13] Sekkarie MA, Moss AH. Withholding and withdrawing dialysis: the role of physician specialty and education and patient functional status. Am J Kidney Dis. 1998;31(3):464–72. 9506683 10.1053/ajkd.1998.v31.pm9506683

[14] Friedman E. Suspected Subterfuge in Proposing Kidney Donor In: Friedman E, editor. Legal and Ethical Concerns in Treating Kidney Failure. Legal and Ethical Concerns in Medicine 1: Springer Netherlands; 2000 p. 143–8.

[15] Phillips B, Ball C, Sackett D, Badenoch D, Straus S, Haynes B, et al. Levels of Evidence Oxford www.cebm.net; 2009.

[16] Grönlund CEF, Söderberg AI, Zingmark KM, Sandlund SM, Dahlqvist V. Ethically difficult situations in hemodialysis care–Nurses’ narratives. Nursing Ethics. 2014 10.1177/0969733014542677 25106455

[17] Grönlund C, Dahlqvist V, Soderberg A. Feeling trapped and being torn: Physicians' narratives about ethical dilemmas in hemodialysis care that evoke a troubled conscience. BMC Medical Ethics. 2011;12(1):8 10.1186/1472-6939-12-8 21569295 PMC3104380

[18] Schicktanz S, Schweda M, Wynne B. The ethics of 'public understanding of ethics'—why and how bioethics expertise should include public and patients' voices. Medicine, health care, and philosophy. 2012;15(2):129–39. Epub 2011/03/31. 10.1007/s11019-011-9321-4 ; PubMed Central PMCID: PMCPmc3319876. 21448745 PMC3319876

[19] Musschenga AW. Empirical ethics, context-sensitivity, and contextualism. The Journal of medicine and philosophy. 2005;30(5):467–90. Epub 2005/11/12. 10.1080/03605310500253030 . 16282140

[20] Visser A, Dijkstra GJ, Kuiper D, de Jong PE, Franssen CF, Gansevoort RT, et al Accepting or declining dialysis: considerations taken into account by elderly patients with end-stage renal disease. J Nephrol. 2009;22(6):794–9. 19967659

[21] Agar JW, Mahadevan K, Knight R, Antonis ML, Somerville CA. 'Flexible' or 'lifestyle' dialysis: is this the way forward? Nephrology (Carlton, Vic). 2005;10(5):525–9. Epub 2005/10/14. 10.1111/j.1440-1797.2005.00473.x . 16221107

[22] Stack AG, Messana JM. Renal replacement therapy in the elderly: medical, ethical, and psychosocial considerations. Adv Ren Replace Ther. 2000;7(1):52–62. 10672917 10.1016/s1073-4449(00)70006-9

[23] Schatell D, Alt PS. Dialysis options education: is 'modality neutrality' fair to patients? Nephrology news & issues. 2008;22(13):24, 6–7. Epub 2009/01/20. . 19149313

[24] Kee F, Reaney E, Savage G, O'Reilly D, Patterson C, Maxwell P, et al Are gatekeepers to renal services referring patients equitably? J Health Serv Res Policy. 2007;12(1):36–41. 17244396 10.1258/135581907779497530

[25] Tostivint I. [New ethical issues around dialysis. To dialyze or not to dialyze, that is not the question]. Presse medicale (Paris, France: 1983). 2007;36(12 Pt 2):1875–81. Epub 2007/09/18. 10.1016/j.lpm.2007.04.040 . 17870277

[26] Marczewski K, Przygoda-Dreher A. End stage renal disease by patients with malignancy—ethical problems. Adv Med Sci. 2006;51:127–32. 17357292

[27] Draper H. Ethical aspects of withdrawing/withholding renal replacement therapies on patients in acute renal failure in an intensive care unit. EDTNA/ERCA journal (English ed). 2002;Suppl 2:39–42. Epub 2002/10/10. . 12371721 10.1111/j.1755-6686.2002.tb00255.x

[28] Moss AH. Shared decision-making in dialysis: the new RPA/ASN guideline on appropriate initiation and withdrawal of treatment. Am J Kidney Dis. 2001;37(5):1081–91. 11325693 10.1016/s0272-6386(05)80027-7

[29] Kujdych N, Lowe DA, Sparks J, Dottes A, Crook ED. Dignity or denial? Decisions regarding initiation of dialysis and medical therapy in the institutionalized severely mentally retarded. Am J Med Sci. 2000;320(6):374–8. 11149549 10.1097/00000441-200012000-00004

[30] Hermsen M, van der Donk M. Nurses' moral problems in dialysis. Nurs Ethics. 2009;16(2):184–91. Epub 2009/02/25. 10.1177/0969733008100078 . 19237472

[31] Badzek LA, Cline HS, Moss AH, Hines SC. Inappropriate use of dialysis for some elderly patients: nephrology nurses' perceptions and concerns. Nephrology nursing journal: journal of the American Nephrology Nurses' Association. 2000;27(5):462–70; discussion 71–2. Epub 2006/05/03. . 16649321

[32] Moss AH. Recommendations regarding conflict resolution and forgoing dialysis in special patient populations. Nephrology news & issues. 2001;15(13):51–4. Epub 2002/07/09. . 12099239

[33] Davidson IJ. Renal impact of fluid management with colloids: a comparative review. European journal of anaesthesiology. 2006;23(9):721–38. Epub 2006/05/26. 10.1017/s0265021506000639 . 16723059

[34] Burguet A, Abraham-Lerat L, Cholley F, Champion G, Bouissou F, Andre JL. [Terminal and pre-terminal chronic renal insufficiency in newborns in French neonatal intensive care units: survey of the French pediatric nephrologic society of resuscitation and emergency]. Arch Pediatr. 2002;9(5):489–94. 12053542 10.1016/s0929-693x(01)00830-2

[35] Clement R, Chevalet P, Rodat O, Ould-Aoudia V, Berger M. Withholding or withdrawing dialysis in the elderly: the perspective of a western region of France. Nephrol Dial Transplant. 2005;20(11):2446–52. 16115859 10.1093/ndt/gfi012

[36] Zamperetti N, Ronco C, Digito A, Piccinni P, Dan M. Results from ethical international surveys on the management of the continuous renal replacement therapy. Minerva anestesiologica. 1999;65(6):437–9. Epub 1999/07/08. . 10394816

[37] Kjellstrand C, Dossetor JB. Ethical Problems in Dialysis and Transplantation: Springer Netherlands; 1992.

[38] Orr RD. Medical ethics and the faith factor: a handbook for clergy and health-care professionals Grand Rapids, Mich.: William B. Eerdmans Pub. Co.; 2009.

[39] Maher JF. Replacement of renal function by dialysis: a textbook of dialysis Dordrecht; Boston: Kluwer Academic Publishers; 1989.

[40] Coustasse A. Hospital costs and clinical characteristics of continuous renal replacement therapy patients: a continuous ethical dilemma. J Hosp Mark Public Relations. 2008;18(2):187–95. 10.1080/15390940802232481 19042868

[41] Davison SN, Holley JL. Ethical issues in the care of vulnerable chronic kidney disease patients: the elderly, cognitively impaired, and those from different cultural backgrounds. Adv Chronic Kidney Dis. 2008;15(2):177–85. 10.1053/j.ackd.2008.01.004 18334244

[42] White Y, Fitzpatrick G. Dialysis: prolonging life or prolonging dying? Ethical, legal and professional considerations for end of life decision making. Edtna Erca J. 2006;32(2):99–103. 16898103 10.1111/j.1755-6686.2006.tb00460.x

[43] Conneen S, Tzamaloukas AH, Adler K, Keller LK, Bordenave K, Murata GH. Withdrawal from dialysis: ethical issues. Dialysis & transplantation. 1998;27(4):200, 2–4. Epub 2005/12/24. . 16370109

[44] Markowitz AJ, Rabow MW. Practical considerations in dialysis withdrawal: "to have that option is a blessing". Jama. 2003;290(6):815 12915435 10.1001/jama.290.6.815

[45] Miller RB. Philosophical underpinnings of and practical considerations in the withdrawal of patients from chronic dialysis. Nephrol News Issues. 2001;15(8):18–22. 12099226

[46] Cooper L. Are patient rights being held hostage? When the final decision is no. Nephrol News Issues. 2001;15(7):43–7. 12098993

[47] Schmidt RJ, Moss AH. Dying on dialysis: the case for a dignified withdrawal. Clinical journal of the American Society of Nephrology: CJASN. 2014;9(1):174–80. Epub 2013/08/24. 10.2215/cjn.05730513 ; PubMed Central PMCID: PMCPmc3878704. 23970133 PMC3878704

[48] Rodriguez Jornet A, Garcia Garcia M, Hernando P, Ramirez Vaca J, Padilla J, Ponz E, et al [Patients with end-stage chronic renal insufficiency on programmed withdrawal from dialysis]. Nefrologia. 2001;21(2):150–9. 11464648

[49] Singer PA. Nephrologists' experience with and attitudes towards decisions to forego dialysis. The End-Stage Renal Disease Network of New England. Journal of the American Society of Nephrology: JASN. 1992;2(7):1235–40. . 1591363 10.1681/ASN.V271235

[50] Schaefer K, von Herrath D, Faust J, Röhrich B. The very elderly dialysis patient: Indication and discontinuation of dialysis. Int Urol Nephrol. 2002;34(4):573–6. 10.1023/A:1025692020673 14577507

[51] Wolfe WA. Achieving equity in referrals for renal transplant evaluations with African-American patients: the role of nephrology social workers. Soc Work Health Care. 2003;37(3):75–87. 14526877 10.1300/J010v37n03_05

[52] Will T, Saudan P, Droulez MG, Soulignac R, Martin PY. [Relationship and dependency in a hemodialysis unit]. Nephrol Ther. 2008;4(5):320–4. 10.1016/j.nephro.2007.10.002 18243086

[53] Davidson SN, Murtagh FE, Higginson IJ. Methodological considerations for end-of-life research in patients with chronic kidney disease. J Nephrol. 2008;21(3):268–82. 18587713

[54] Hou S. Expanding the kidney donor pool: ethical and medical considerations. Kidney Int. 2000;58(4):1820–36. 11012922 10.1046/j.1523-1755.2000.00345.x

[55] Moss AH. New guideline describes nephrology community consensus on withholding and withdrawing dialysis. Recommendations regarding withdrawing dialysis. 2. Nephrology news & issues. 2001;15(12):58–61. Epub 2002/07/09. . 12099182

[56] Gomez Campdera FJ, Luno J, Garcia de Vinuesa MS, Valderrabano F. [Dialysis inclusion criteria and early mortality]. Nefrologia: publicacion oficial de la Sociedad Espanola Nefrologia. 2001;21(2):218–20. . 11464659

[57] Friedman EA. Stressful ethical issues in uremia therapy. Kidney international Supplement. 2010;(117):S22–32. Epub 2010/11/03. 10.1038/ki.2010.190 . 20671740

[58] Svantesson M, Anderzen-Carlsson A, Thorsen H, Kallenberg K, Ahlstrom G. Interprofessional ethics rounds concerning dialysis patients: staff's ethical reflections before and after rounds. Journal of medical ethics. 2008;34(5):407–13. Epub 2008/05/02. 10.1136/jme.2007.023572 . 18448727

[59] Rinehart A. Beyond the futility argument: the fair process approach and time-limited trials for managing dialysis conflict. Clinical journal of the American Society of Nephrology: CJASN. 2013;8(11):2000–6. Epub 2013/07/23. 10.2215/cjn.12191212 ; PubMed Central PMCID: PMCPmc3817917. 23868900 PMC3817917

[60] Jones JW, McCullough LB. Extending life or prolonging death: when is enough actually too much? Journal of vascular surgery. 2014;60(2):521–2. Epub 2014/07/30. 10.1016/j.jvs.2014.05.054 . 25064329

[61] Thorsteinsdottir B, Swetz KM, Tilburt JC. Dialysis in the frail elderly—a current ethical problem, an impending ethical crisis. Journal of general internal medicine. 2013;28(11):1511–6. 10.1007/s11606-013-2494-1 23686511 PMC3797329

[62] Svantesson M, Lofmark R, Thorsen H, Kallenberg K, Ahlstrom G. Learning a way through ethical problems: Swedish nurses' and doctors' experiences from one model of ethics rounds. Journal of medical ethics. 2008;34(5):399–406. Epub 2008/05/02. 10.1136/jme.2006.019810 . 18448726

[63] Friedman MB, Friedman EA. Strength and compassion in kidney failure: writings of Mildred (Barry) Friedman, professional kidney patient Dordrecht; Boston: Kluwer Academic Publishers; 1998.

[64] Watson S. End-of-life issues in renal medicine. Clinical medicine (London, England). 2005;5(6):643–5. Epub 2006/01/18. 16411363. 10.7861/clinmedicine.5-6-643 PMC4953147 16411363

[65] Howard CS, Teitelbaum I. Renal replacement therapy in patients with chronic liver disease. Semin Dial. 2005;18(3):212–6. 15934968 10.1111/j.1525-139X.2005.18315.x

[66] Jansen DL, Rijken M, Heijmans M, Boeschoten EW. Perceived autonomy and self-esteem in Dutch dialysis patients: the importance of illness and treatment perceptions. Psychol Health. 2010;25(6):733–49. 10.1080/08870440902853215 20204947

[67] Muthalagappan S, Johansson L, Kong WM, Brown EA. Dialysis or conservative care for frail older patients: ethics of shared decision-making. Nephrology, dialysis, transplantation: official publication of the European Dialysis and Transplant Association—European Renal Association. 2013;28(11):2717–22. Epub 2013/06/22. 10.1093/ndt/gft245 . 23787549

[68] Lehoux P, Richard L, Pineault R, Saint-Arnaud J. Delivery of high-tech home care by hospital-based nursing units in Quebec: clinical and technical challenges. Nursing leadership (Toronto, Ont). 2006;19(1):44–55. Epub 2006/04/14. . 16610297 10.12927/cjnl.2006.18048

[69] Tovazzi ME, Mazzoni V. Personal paths of fluid restriction in patients on hemodialysis. Nephrology nursing journal: journal of the American Nephrology Nurses' Association. 2012;39(3):207–15. . 22866360

[70] Coyne DW. Managing anemia in for-profit dialysis chains: when ethics and business conflict. Semin Dial. 2009;22(1):18–21. 10.1111/j.1525-139X.2008.00531.x 19250444

[71] Williams ME, Kitsen J. The involuntarily discharged dialysis patient: conflict (of interest) with providers. Adv Chronic Kidney Dis. 2005;12(1):107–12. 15719341 10.1053/j.ackd.2004.10.010

[72] Shaldon S. Conflict of interest. Nephrology, dialysis, transplantation: official publication of the European Dialysis and Transplant Association—European Renal Association. 2004;19(11):2928 Epub 2004/10/22. 10.1093/ndt/gfh396 . 15496576

[73] Garg AX, Blake PG, Clark WF, Clase CM, Haynes RB, Moist LM. Association between renal insufficiency and malnutrition in older adults: Results from the NHANES III. Kidney international. 2001;60(5):1867–74. 11703605 10.1046/j.1523-1755.2001.00001.x

[74] Tweed AE, Ceaser K. Renal replacement therapy choices for pre-dialysis renal patients. Br J Nurs. 2005;14(12):659–64. 16010217 10.12968/bjon.2005.14.12.18287

[75] Thorsteinsdottir B, Swetz KM, Feely MA, Mueller PS, Williams AW. Are there alternatives to hemodialysis for the elderly patient with end-stage renal failure? Mayo Clinic proceedings. 2012;87(6):514–6. Epub 2012/06/09. 10.1016/j.mayocp.2012.02.016 ; PubMed Central PMCID: PMCPmc3498386. 22677071 PMC3498386

[76] Stack AG, Martin DR. Association of patient autonomy with increased transplantation and survival among new dialysis patients in the United States. Am J Kidney Dis. 2005;45(4):730–42. 15806476 10.1053/j.ajkd.2004.12.016

[77] Cronin AJ. Allowing autonomous agents freedom. J Med Ethics. 2008;34(3):129–32. 10.1136/jme.2007.023580 18316449

[78] Weinman J, Petrie KJ, Moss-morris R, Horne R. The illness perception questionnaire: A new method for assessing the cognitive representation of illness. Psychology & health. 1996;11(3):431–45. 10.1080/08870449608400270

[79] Shirahama M. [Dealing with aged patients with kidney failure who refuse dialysis treatment]. Nihon Naika Gakkai Zasshi. 2007;96(2):377–80. 17370605 10.2169/naika.96.377

[80] Riley JB Jr., Pristave RJ. Patient's rights in receiving or rejecting dialysis care. Nephrol News Issues. 2001;15(9):49–51. 12099187

[81] Moss AH. Ethical principles and processes guiding dialysis decision-making. Clinical journal of the American Society of Nephrology: CJASN. 2011;6(9):2313–7. Epub 2011/09/08. 10.2215/cjn.03960411 . 21896833

[82] Holley JL, Davison SN, Moss AH. Nephrologists' changing practices in reported end-of-life decision-making. Clin J Am Soc Nephrol. 2007;2(1):107–11. 17699394 10.2215/CJN.03080906

[83] Grönlund CE, Dahlqvist V, Soderberg AI. Feeling trapped and being torn: physicians' narratives about ethical dilemmas in hemodialysis care that evoke a troubled conscience. BMC Med Ethics. 2011;12:8 Epub 2011/05/17. 10.1186/1472-6939-12-8 ; PubMed Central PMCID: PMCPmc3104380. 21569295 PMC3104380

[84] Davison SN, Jhangri GS, Holley JL, Moss AH. Nephrologists' reported preparedness for end-of-life decision-making. Clinical journal of the American Society of Nephrology: CJASN. 2006;1(6):1256–62. 10.2215/CJN.02040606 . 17699356

[85] Romer G, Stavenow K, Baldus C, Bruggemann A, Claus B, Riedesser P. [How children experience a parent being chronically ill: a qualitative analysis of interviews with children of hemodialysis patients]. Prax Kinderpsychol Kinderpsychiatr. 2006;55(1):53–72. 16471414

[86] Moss AH. New guideline describes. Nephrology community consensus on withholding and withdrawing dialysis. 7. Nephrology news & issues. 2001;15(11):50, 5–6, 7. Epub 2002/07/09. . 12098983

[87] Weisbord SD, Fried LF, Mor MK, Resnick AL, Kimmel PL, Palevsky PM, et al Associations of race and ethnicity with anemia management among patients initiating renal replacement therapy. Journal of the National Medical Association. 2007;99(11):1218–26. Epub 2007/11/21. ; PubMed Central PMCID: PMCPmc2574315. 18020096 PMC2574315

[88] Friedman EA. Must we treat noncompliant ESRD patients? Semin Dial. 2001;14(1):23–7. 11208035 10.1046/j.1525-139x.2001.00009.x

[89] Levinsky NG, Yu W, Ash A, Moskowitz M, Gazelle G, Saynina O, et al Influence of age on Medicare expenditures and medical care in the last year of life. JAMA. 2001;286(11):1349–55. . 11560540 10.1001/jama.286.11.1349

[90] Moseley KL, Kershaw DB. African American and white disparities in pediatric kidney transplantation in the United States—unfortunate or unjust? Camb Q Healthc Ethics. 2012;21(3):353–65. 10.1017/S0963180112000072 22624538

[91] Donnelly P, Oman P, Opelz G. Should culture-based gender bias in living donor renal transplantation be discouraged on ethical and graft survival grounds? Transplant Proc. 2001;33(1–2):1882–3. 11267552 10.1016/s0041-1345(00)02735-4

[92] Anantharaman P, Moss AH. Should the medicare ESRD program pay for daily dialysis? An ethical analysis. Advances in chronic kidney disease. 2007;14(3):290–6. Epub 2007/07/03. 10.1053/j.ackd.2007.03.001 . 17603984

[93] Ashwanden C. Quantity versus quality: ethics and provision of renal replacement therapy. EDTNA/ERCA journal (English ed). 2001;27(1):31–3. Epub 2003/02/27. . 12603071 10.1111/j.1755-6686.2001.tb00132.x

[94] Mendelssohn DC. Increasing PD utilization: should suitable patients be forced? Peritoneal dialysis international: journal of the International Society for Peritoneal Dialysis. 2009;29(2):144–6. Epub 2009/03/19. . 19293349

[95] Isaacs R. Ethical implications of ethnic disparities in chronic kidney disease and kidney transplantation. Adv Ren Replace Ther. 2004;11(1):55–8. 14730538 10.1053/j.arrt.2003.10.008

[96] Pinker S. Can Quebec afford dialysis for every 80-year-old patient? CMAJ: Canadian Medical Association journal = journal de l'Association medicale canadienne. 2000;162(2):243 Epub 2000/02/16. ; PubMed Central PMCID: PMCPmc1232280. 10674063 PMC1232280

[97] Spital A. Ethical issues in dialysis. Managing noncompliant dialysis patients. Seminars in dialysis. 2001;14(1):22 Epub 2001/02/24. . 11208034 10.1046/j.1525-139x.2001.00008.x

[98] Holmes D, Perron AM, Savoie M. Governing therapy choices: power/knowledge in the treatment of progressive renal failure. Philos Ethics Humanit Med. 2006;1:12 17144913 10.1186/1747-5341-1-12 PMC1693913

[99] Rehman R, Schmidt RJ, Moss AH. Ethical and legal obligation to avoid long-term tunneled catheter access. Clinical journal of the American Society of Nephrology: CJASN. 2009;4(2):456–60. Epub 2009/01/23. 10.2215/cjn.03840808 . 19158368

[100] Moss AH. Shared Decision Making in dialysis: a new clinical practice guideline to assist with dialysis-related ethics consultations. J Clin Ethics. 2001;12(4):406–14. 12026747

[101] Holley JL, Carmody SS, Moss AH, Sullivan AM, Cohen LM, Block SD, et al The need for end-of-life care training in nephrology: national survey results of nephrology fellows. American journal of kidney diseases: the official journal of the National Kidney Foundation. 2003;42(4):813–20. Epub 2003/10/02. . 14520633 10.1016/s0272-6386(03)00868-0

[102] Ang A, Loke PC, Campbell AV, Chong SA. Live or let die: ethical issues in a psychiatric patient with end-stage renal failure. Ann Acad Med Singapore. 2009;38(4):370–4. 19434342

[103] Bunchman TE. The ethics of infant dialysis. Peritoneal dialysis international: journal of the International Society for Peritoneal Dialysis. 1996;16 Suppl 1:S505–8. Epub 1996/01/01. . 8728257

[104] Oneschuk D, Fainsinger R. Medical and ethical dilemmas when an advanced cancer patient discontinues dialysis. Journal of palliative care. 2002;18(2):123–6. Epub 2002/08/08. . 12164100

[105] Davison SN. The ethics of end-of-life care for patients with ESRD. Clinical journal of the American Society of Nephrology: CJASN. 2012;7(12):2049–57. Epub 2012/09/22. 10.2215/cjn.03900412 . 22997341

[106] Hashmi A, Moss AH. Treating difficult or disruptive dialysis patients: practical strategies based on ethical principles. Nature clinical practice Nephrology. 2008;4(9):515–20. Epub 2008/07/10. 10.1038/ncpneph0877 . 18612329

[107] Balint J. There is a duty to treat noncompliant patients. Semin Dial. 2001;14(1):28–31. 11208036 10.1046/j.1525-139x.2001.00010.x

[108] Bernardini J. Ethical issues of compliance/adherence in the treatment of hypertension. Adv Chronic Kidney Dis. 2004;11(2):222–7. 15216495 10.1053/j.arrt.2004.01.003

[109] Lantos JD, Warady BA. The evolving ethics of infant dialysis. Pediatric nephrology (Berlin, Germany). 2013;28(10):1943–7. Epub 2012/11/08. 10.1007/s00467-012-2351-1 ; PubMed Central PMCID: PMCPmc3626731. 23131864 PMC3626731

[110] Bouissou F. [Kidney failure in the newborn]. Arch Pediatr. 2006;13(6):723–5. Epub 2006/05/16. doi: S0929-693X(06)00199-0 [pii] 10.1016/j.arcped.2006.03.105 . 16697593

[111] Winearls CG. In the wake of progress—ethical problems of renal failure treated by dialysis. Clinical medicine (London, England). 2006;6(1):76–80. Epub 2006/03/09. . 16521361 10.7861/clinmedicine.6-1-76 PMC4954439

[112] Bagon JA, Vernaeve H, De Muylder X, Lafontaine JJ, Martens J, Van Roost G. Pregnancy and dialysis. American journal of kidney diseases: the official journal of the National Kidney Foundation. 1998;31(5):756–65. Epub 1998/05/20. . 9590184 10.1016/s0272-6386(98)70060-5

[113] Romao JE Jr., Luders C, Kahhale S, Pascoal IJ, Abensur H, Sabbaga E, et al Pregnancy in women on chronic dialysis. A single-center experience with 17 cases. Nephron. 1998;78(4):416–22. Epub 1998/05/15. . 9580542 10.1159/000044970

[114] Ralph C. Pregnancy in a hemodialysis patient with an ethical/cultural challenge. Cannt J. 2000;10(1):35–8. 15719603

[115] Di Benedetto A, Buono A, Cappabianca F, Marinelli G. Dialysis in elderly patients. Lancet. 2001;358(9291):1463 Epub 2001/11/14. 10.1016/s0140-6736(01)06515-1 . 11705537

[116] Ortega F, Gomez E, Baltar J, Rebollo P. [Controversies in nephrology: dialysis in the elderly]. Nefrologia: publicacion oficial de la Sociedad Espanola Nefrologia. 2001;21(4):332–6. Epub 2002/01/31. . 11816505

[117] Cupa D, Riazuelo H, Causeret C, Gourdon ML, Pirlot G. [Patients on dialysis and the psychological aging process]. Nephrol Ther. 2009;5(2):102–8. 10.1016/j.nephro.2008.04.004 18678540

