# Supplementary material for: The Full Spectrum of Clinical Ethical Issues in Kidney Failure. Findings of a Systematic Qualitative Review
Source: PLoS One. 2016 Mar 3;11(3):e0149357. doi: 10.1371/journal.pone.0149357 (PMC4777282; doi:10.1371/journal.pone.0149357)
Supplement: S1 Table — (DOCX) [file pone.0149357.s001.docx]

**S1 Table. Details on the search strategy.**

| Database | Medline |
| --- | --- |
| **Search expression** | ((((Ethics[Mesh]) OR ethical[ti]) OR ethics[ti])) AND (((((Renal Dialysis[Mesh]) OR Kidney Failure, Chronic[ti]) OR Renal Insufficiency[ti]) OR Kidney Failure, Chronic[Mesh]) OR Renal Insufficiency[Mesh]) |
| **Date** | January 2015 |
| Filter | Language restrictions: English and German |
| Hits | 233 |
